# The impact of EU public procurement regulations on tenders in Spain: a study with adalimumab

**DOI:** 10.3389/fphar.2024.1447324

**Published:** 2024-09-19

**Authors:** F. J. Esplugues, I. Andújar, J. V. Esplugues

**Affiliations:** ^1^ Department of Pharmacology, University of Valencia, Valencia, Spain; ^2^ CIBEREHD (Centro de Investigación Biomédica en Red de Enfermedades Hepáticas y Digestivas), Madrid, Spain

**Keywords:** tender of pharmaceuticals, public procurement, biosimilars, biologicals, adalimumab, cost-containment, health expenditure

## Abstract

**Introduction:**

Pharmaceutical spending accounts for a significant portion of public healthcare budgets. To manage these costs, EU countries implement various cost-containment policies, including competitive tendering for pharmaceuticals. This study examines the impact of EU public procurement regulations on medication procurement practices.

**Methods:**

A search for all published tenders of adalimumab in Spain from 2018 to 2024 in the Spanish Public Sector Procurement Database, a period that coincides with the implementation of European legislation and the emergence of adalimumab biosimilars. All available documentation for each tender was reviewed, including the tender offer, technical specifications, specific administrative clauses, appointments of evaluation commissions, supporting memorandum, and evaluation reports.

**Results and Discussion:**

Our findings reveal substantial price reductions following the introduction of adalimumab biosimilars, yet highlight significant variability in tender criteria and practices across different regions. Despite adherence to EU directives, the inconsistent application of economic and non-economic factors and an erratic criteria concerning price undermine the intended balance of quality and cost, complicating procurement processes and potentially affecting the availability of a given treatment for patients.

## 1 Introduction

Health expenditure in the European Union (EU) approached 7.7% of Gross National Product (GDP) in 2022 (Eurostat, 2024). Despite their varied characteristics, all EU countries predominantly operate under a public healthcare system. Within this system, pharmaceutical spending is a major budget item, ranging from 6.4% of total public healthcare expenses in Denmark to 26.9% in Greece in 2022 (OECD, 2024). These figures reflect an ongoing upward trend that has persisted for over 3 decades, and shows no signs of abating, due to an aging population and rising costs associated with new medical technologies and drugs (Ciulla et al., 2023). Given the substantial contribution of medicines to total healthcare costs, there is a continuous effort to implement cost-containing policies. Most countries already regulate pharmaceutical prices and strive to rationalize the demand for medication consumption.

Competitive tendering has become a pivotal strategy for reducing healthcare costs, proving particularly effective when alternative medications, such as generic drugs, are available. This procurement process is structured to select the most cost-efficient supplier, aiming to minimize and stabilize purchasing prices throughout the duration of a specified contract period (Ehlers et al., 2022). By fostering competition among suppliers and shifting market influence towards the purchaser, competitive tendering usually leads to significant reductions in purchase prices (Dranitsaris et al., 2017). However, while this strategy successfully lowers acquisition costs, it also introduces potential risks that can adversely affect the healthcare system. These risks stem from suboptimal tendering practices by policymakers and buyers, which may include a lack of transparency in how tenders are awarded, inconsistent procedures across different tenders, and unclear or poorly defined criteria for selecting winners (Barbier et al., 2021). Often, there is an excessive focus on selecting the lowest-priced offer, which can overlook the quality and sustainability of the healthcare products (Simoens and Cheung, 2020). Additionally, the practice of awarding the contract to a single winner can suppress competition and reduce supplier diversity, which might lead to monopolistic behaviors and impact drug availability (Dranitsaris et al., 2017; Németh et al., 2023). Moreover, poorly managed tender processes can trigger undesirable responses from pharmaceutical companies. These companies, concerned with diminishing returns on investment, may decide to withdraw from markets or cut back on their commitment to research and development, which can lead to drug shortages and negatively impact drug innovation (Dranitsaris et al., 2017; Barbier et al., 2021; Simoens and Cheung, 2020; Németh et al., 2023).

The EU emphasizes that all public tendering should adhere to the principles of transparency, equality, non-discrimination and the pursuit of the optimal “quality/price ratio.” To implement these concepts any public authority within the EU that awards a contract for works, supplies, or services surpassing predefined financial thresholds should conform with the Public Procurement Directive 2014/24/EU “on public procurement and repealing Directive 2004/18/EC” and Directive 2014/23/EU “on the award of concession contracts” (European Union, 2014b; European Union, 2014a; European Union, 2004). Additionally, such acquisitions are to be documented in a public registry detailing the main features of the tender leading to the procurement. However, the range of health and non-health-related public purchases is vast, and no single regulation can realistically cover all the specificities needed to define an ideal “quality/price ratio.” Our study explores the impact of the European regulatory framework on public medication procurement practices. Numerous studies have investigated various aspects of drug procurement tenders across European countries, primarily focusing on their effects on cost reduction, drug substitution, and prescribing practices (Ehlers et al., 2022; Simoens and Cheung, 2020; García-Altés et al., 2023; Messori et al., 2020; Wilsdon et al., 2020; Dylst et al., 2011). However, these studies handle complex tenders featuring multiple pharmacological options and diverse technical specifications, leading to intricate analyses that obscure practical conclusions, and often rely on subjective data collection methods such as surveys or interviews with stakeholders in pharmaceutical procurement. To overcome this challenge and simplify the task, we have focused on the case of public tenders for adalimumab in Spain, which adopted European legislation in 2017 (Ley 9, 2017), and based our study on concrete and objective data obtained directly from Spanish public procurement registers.

Biologic medicines, a type of drug derived from living organisms, represent a significant part of pharmaceutical expenses due to their effectiveness in treating complex conditions. In Europe, they account for 35% of medication spending at list prices and their cost has been growing at almost twice the pace of non-biologic medicines over the past 5 years (Troein et al., 2022). Adalimumab is a biologic drug classified as a monoclonal antibody that is administered via subcutaneous injection. It specifically targets and inhibits tumor necrosis factor alpha (TNF-α), a pro-inflammatory cytokine involved in the pathogenesis of various autoimmune diseases. By blocking the action of TNF-α, it is effective in treating conditions such as rheumatoid arthritis, psoriatic arthritis, ankylosing spondylitis, Crohn’s disease, and ulcerative colitis (Lu et al., 2021). This drug has occupied a significant niche in the treatment of these diseases, having been the world’s top-selling medication by economic value for nearly a decade (Gibbons et al., 2023). It has been selected for study because its patent expired in Europe in 2018, coinciding with the new European regulatory framework and its implementation in Spain. Interest in adalimumab has increased since its patent expired, as the original compound now faces competition from biosimilars (Lu et al., 2021). The European Medicines Agency (EMA) was the first regulatory agency to approve a biosimilar of a TNF-α blocker in 2013 and has continued to authorize these drugs since then. The EMA defines biosimilars not as generics, but as biologic replicas that, under a new and comprehensive review process, must demonstrate “biosimilarity” to the original compound and can only be prescribed for the same indications (European Medicines Agency, 2012; Mellstedt, 2013). Thus, once their “biosimilarity” is established, there are only a limited number of technical variables (such as dosage, or minor changes in solvent and/or the injection device) among the various marketed adalimumab products that can be considered in a tender to respond to the EU-required balance of “quality” *versus* “cost” (European Union, 2014b; European Union, 2014a; Andújar et al., 2018). By 2022, there were 10 biosimilars of adalimumab authorized in Europe, which represents more than double the number of competitors compared to other biologics with biosimilars on the market (with an average of 3.8 competitors each) (Ehlers et al., 2022).

Positioned as the European Union’s fourth-largest country in terms of population and economy, Spain presents a unique healthcare context. It integrates a central governing body, the Ministry of Health, which approves drugs and their pricing, with 17 autonomous geographical entities, each possessing significant autonomy in healthcare management, including medication procurement. Our research investigates the adalimumab procurement patterns in Spain from 2018 to 2024, a period that coincides with the beginning of the implementation of European legislation and the emergence of adalimumab competitors under the new concept of “biosimilarity.” Within this narrow scenario, we aimed to uncover the practices, peculiarities, and compliance with the aims of the European directive among different actors (different contracting authorities and suppliers) operating within a similar legal framework. Our evaluation provides a real-time picture of the regulatory impact of European legislation on public pharmaceutical acquisitions.

## 2 Materials and methods

A search for all published tenders of adalimumab in Spain from 1 January 2018, to 30 April 2024 was carried out. The search was conducted in the Spanish Public Sector Procurement Database (https://contrataciondelestado.es/, last accessed on 7 June 2024 (Contratacion, 2024)), a platform that aggregates contracts carried out at the state and autonomous community levels, using the Common Procurement Vocabulary (CPV) 33600000 “pharmaceutical products” (European Parliament and Council of the European Union, 2002) followed by an individualized search for the words “adalimumab and/or biosimilar”). Some autonomous communities (Madrid, Catalonia, Andalusia, Basque Country, Galicia, La Rioja and Navarre) have their own platforms that were also searched for the term “adalimumab”, and duplicates were discarded. Bids that incorporated adalimumab alongside other medications were included in the search, but the study focuses only on tenders of adalimumab with an estimated contract value exceeding €1,000,000. Negotiated contracts without advertising, and thus without tendering, such as those focused on the purchase of the original adalimumab for the continuity of existing treatments, were not incorporated into this study.

All available documentation for each tender was reviewed, including the tender offer, technical specifications, specific administrative clauses, appointments of evaluation commissions, supporting memorandum, and evaluation reports. Information regarding the dates of publication and tender, number of successful bidders, the technical requirements demanded by each tender, and the specific selection criteria detailing economic and non-economic factors, was also analyzed. Adapting a previously suggested model to facilitate best-value biological selection (Barbier et al., 2022), all non-economic factors considered in every analyzed tender have been amalgamated in Table 1 under three general headings: (a) product-driven criteria (which include technical product features and licensed therapeutic indications); (b) service-driven criteria (consisting of supply conditions, value-added services, and environment and sustainability criteria); and (c) patient-driven criteria (containing factors related to assisting adalimumab self-injection, such as ease of use or patient support/learning programs).

**TABLE 1:** Items enlisted under each category of non-economic factors.

Patient-driven criteria
Systems facilitating handling for dispensing/reconstitution/administration
Presence of a system to ensure safety in medication handling
Free delivery of a treatment starter kit containing support materials and accessories for the proper use of administration devices and to promote treatment adherence
Patient education and support program
Needle gauge (the smaller, the better)
Presentation volume (lower volume is preferable)

Product-driven criteria
Absence of excipients with mandatory declaration
Absence of latex
Absence of monohydrated citric acid or sodium citrate
Visual differentiation between the various presentations of the medication
Packaging and labeling of transport boxes
Light protection measures
Two-dimensional barcode on the primary packaging
Datamatrix containing the national identification code, batch number and expiration date
Suitability for automated pharmacy distribution systems (appropriate packaging size)
Indication of the administration route
Packaging characteristics (unbreakable and contamination-free, either covered with plastic or cleaned)
Pharmaceutical form
Free delivery of isothermal transport devices
Free delivery of waste collection containers to facilitate the disposal of injectable devices used by patients at home
Validity/expiry/stability period
Number of available presentations in the market.
Number of approved indications

Service-driven criteria
Commitment to maintain minimum medication stock equivalent to 2 months of consumption, ensuring adequate continuity of supply throughout the duration of the contract
Commitment to uninterrupted supply in a timely manner (absence of stockouts)
Commitment to maximum reliability in order management
Commitment to providing of urgent delivery services within 24 h
Compliance with emergency and disaster preparedness services
Delivery time for regular orders (between 24–72 h according to the tender)
Cold chain traceability
Sending of valued delivery notes
Single-dose dispensing
No minimum order requirement
100% discount on the induction treatment, matching its value to that of the maintenance treatment
100% discount on the intensification treatment in case of secondary failure, matching its value to that of the maintenance treatment
Replacement of merchandise with identical or equivalent products in cases of expiration, deterioration, or market withdrawal
Absence of precautionary immobilization proceedings for the product in the last 2 years
Availability of a warehouse in the Canary Islands
Operational provider messaging system within the Canary Islands Health Service
Carbon footprint assessment
Other distinctive technical aspects identified by the Promoting Unit that may be of interest to service organizations

## 3 Results and discussion

The present analysis of adalimumab tender processes concentrates on two pivotal aspects: the expiration of the original adalimumab patent in the European market, which facilitated the emergence of adalimumab biosimilars, and the concurrent adaptation to European public procurement regulations within Spain. These regulations underscore the importance of transparency and extend the evaluative criteria of public tenders to include both price and quality. According to the Directive 2014/24/EU of the European Parliament (European Union, 2014b), “criteria to evaluate quality may comprise, for instance, (a) quality, including technical merit, aesthetic and functional characteristics, accessibility, design for all users, social, environmental and innovative characteristics and trading and its conditions; (b) organization, qualification and experience of staff assigned to performing the contract, where the quality of the staff assigned can have a significant impact on the level of performance of the contract; or (c) after-sales service and technical assistance, delivery conditions such as delivery date, delivery process and delivery period or period of completion.” Our dataset encompasses 16 ordinary tenders, a number that may seem limited given that we are dealing with an important and widely used drug during a 6-year period, but is representative of the mammoth bureaucratic effort required for a well-organized tender. 15 of the 16 tenders were generated by 9 individual Autonomous Regions, collectively representing 76% of the Spanish population (47.5 million) and 1 was generated by an institution related to the Ministry of Health (INGESA, “Instituto Nacional de Gestión Sanitaria,” National Institute of Health Management) that is responsible for negotiating a general tender whose contracting framework can be adopted voluntarily by those autonomous communities that prefer not to generate their own specific tenders. In all the cases evaluated, the characteristics of the data available in the Public Sector Procurement Databases was very thorough, incorporating drug and non-drug related features, price, and the composition of the panel evaluating the tender, thus in line with the spirit of EU Directives (European Union, 2014b; European Union, 2014a).

The combined worth of the 16 tenders completed between 2018 and 2024 stands at €528 million, with individual tenders ranging from €7.6 million to €109.6 million and an average estimated value per tender of approximately €33 million. These contracts typically last between three to 5 years, potentially including annual extensions predetermined by contract stipulations. Although we believe that we have accessed all of the published public sector purchasing data for adalimumab in Spain, the Spanish Independent Authority for Fiscal Responsibility (AIREF) (AIREF Autoridad Independiente de Responsabilidad Fiscal, 2020) identified that approximately two-thirds of public procurements of medical equipment and drugs in Spanish hospitals in 2018 did not comply with mandated Spanish and European regulations and were conducted through small contracts or direct purchasing. The situation is likely to have improved in the last 5 years, but there is some preliminary evidence that a substantial number of purchases of health-related items are still being executed outside of standardized procurement channels (AIREF Autoridad Independiente de Responsabilidad Fiscal, 2024). In the case of pharmaceuticals this scenario is likely to be related to the COVID-19 pandemic, the purchase of originator drugs where there is no competition, or the need to cover unexpected interruptions in the supply of medicines. However, we do not believe this compromises the essence of our evaluation. By using the example of adalimumab (high cost, narrow and specific medical indications, limited number of suppliers, a reduced number of technical variables), we believe we have provided a valid snapshot of how Spanish tenders for pharmaceuticals have evolved to comply with EU directives for public procurement (European Union, 2014b; European Union, 2014a).

**FIGURE 1:**
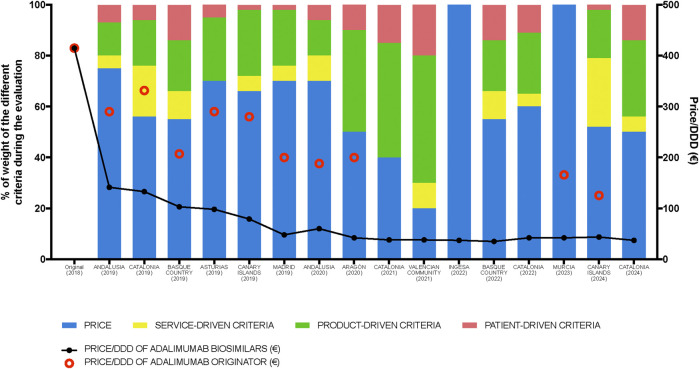
Percentage of weight given to the economic factor and to each category of non-economic factors (defined in Table 1) found in the analyzed tenders. Black dots represent the lowest defined-daily-dose (DDD) price of adalimumab biosimilars (€) (40 mg presentation) offered by the winning tender. Red circles represent the price/DDD (€) of the originator product in those tenders in which it participated. (INGESA: “Instituto Nacional de Gestión Sanitaria”, National Institute of Health Management).

The introduction of biosimilars is known to drive down the prices of original biologics (Dylst and Simoens, 2010; Moorkens et al., 2021a), as evidenced by our case study of adalimumab. In Spain in 2024 there are 7 brands of adalimumab, each with at most three difference dosages, and some differences in injection volumes or pharmaceutical presentations such as prefilled syringe *versus* pen. Prior to the first evaluated tender in December 2018, the lowest price of the original adalimumab was €415 per defined-daily-dose (DDD). This price decreased to €141 in the first tender in 2019, marking a 66% reduction with respect to the initial price. It continued to decrease in subsequent tenders until it stabilized between €35 and €42 by mid-2020, a figure that has remained relatively unchanged since then (Figure 1). During the 6-year period evaluated, the biosimilar that was awarded the tender consistently had a significantly lower price than the originator that competed for the assessed public tenders. Despite the substantial potential for price reduction in earlier tenders due to the prevailing high prices of the drug, which should have given substantial weight to the consideration of cost, the quality factor was also expected to be influential due to initial reservations about biosimilars of monoclonal antibodies when they were first marketed 10 years ago. Concerns included potential immunogenicity, substitution of an ongoing successful treatment with the original molecule, single or multiple switching with different biosimilars, and the prevalence of nocebo effects, among others. These issues led to either a slow uptake in the use of biosimilars or unexpected usage patterns in both Spain and other EU countries (Reuber and Kostev, 2019; Dylst et al., 2014; Moorkens et al., 2016; Moorkens et al., 2021b). However, over time, familiarity with these compounds in the EMA-regulated area has increased, they have gained a higher level of acceptance and confidence in their use has grown (Troein et al., 2022; Kurki et al., 2021). With price reductions now exceeding 85%, and thus little room for further economic savings, our initial hypothesis was two-fold: (a) that in a scenario with limited technical or pharmacological variables, such as that of adalimumab, once costs stabilization was accomplished, considerations regarding the market sustainability of biosimilars (like encouraging competition and maintaining supplier diversity) would become more prevalent; and (b) this maintenance of low prices would lead to a reevaluation of the importance of certain quality factors, for instance longer shelf life, ease of use of the device, or patient-support programs, whose importance would increase over time.

Our premise was proven wrong as the patterns observed in successive tenders did not follow any predictable trends. Indeed, across all tenders, the most prominent feature was a significant difference in the emphasis on the economic factor, specifically price, without a temporal framework to establish correlations and no significant convergence in the appraisal of cost over time. In two of the sixteen tenders evaluated (representing 12.5% of the tenders), price was the only criterion considered. In one of these two cases the tender was not won by the company or companies with the lowest price, but by all those that were below the price threshold established by the call, which was substantially higher than the standard market price at the time and included the price of the originator. This meant that, rather than a proper tender, it was more like an “accreditation process” through which, as long as a maximum price-cap was not exceeded, most commercially available adalimumab products were approved, allowing a specific hospital to choose the adalimumab it desired. In 37.5% of tenders (six out of sixteen), price was the predominant factor, but other elements were also considered in the evaluation. A roughly equal distribution between price and other criteria was found in six of the sixteen tenders (37.5%). Finally, two of the sixteen tenders (12.5%) were awarded predominantly based on criteria other than price. Although this does not reflect total compliance with the European directive, it is more aligned with the spirit of said directive than in other European countries, where it is estimated that over 20% of tenders for biosimilars are awarded based entirely on price (Barbier et al., 2021).

Excluding the two tenders where price was the sole criterion, up to 41 distinct characteristics assessed across the remaining 14 tenders were identified (Table 1). When categorizing the numerous individual characteristics within the three broad headlines previously defined (product-driven, service-driven and patient-driven criteria), product-related features tended to be the most valued (Figure 1). Conducting a deeper analysis or finding coherence in the evaluations was challenging due to the significant disparity among the various tenders regarding which precise items were required and the value attributed to each specific one. Some of the items seem sound, such as the demand for a full range of dosages or that packaging is suitable for the characteristics of the hospitals’ automated distribution system, or the need for a warehouse to ensure continuity of supply on an island. But others are more difficult to categorize. For example, the emphasis on the rapid delivery of medication, tagged under “service-driven category” was highlighted as “critical” in four tenders, while it was overlooked in the others. This is likely due to standing Spanish legislation that mandates a 24-h delivery period as a bidding prerequisite for any pharmaceutical tenders, rendering this demand unnecessary or repetitive. This variability, present even within tenders from the same autonomous region, precludes the ability to discern any uniform pattern or common evaluative criteria across the tenders. This situation is not unusual, and indeed appears to be common across Europe: a study carried out to assess the extent and impact of value-added services in biosimilar tendering in several other European countries (Norway, Italy, England, France and Ireland) concluded that there was a similar lack of standardized criteria. This study also emphasized the need for greater consistency in both the criteria included and the weighting applied to quality items in biosimilar tendering procedures (Simoens and Cheung, 2020)

Thirteen of the evaluated tenders were designed to be awarded to a single winner (Table 2), which aligns with the documented European situation for other biosimilars (European Parliament and Council of the European Union, 2002). However, among the tenders initially designed to have one awardee, eight of them allowed the purchase adalimumab from other companies that did not win the initial tender but had exceeded a minimum level of requirements, provided it could be justified for clinical reasons (i.e., the possibility of medical personnel being able to choose) or logistics (i.e., avoidance of supply shortages). A single-winner tender strategy typically achieves substantial discounts, particularly when dealing with high product volumes, as is the case in the present analysis of adalimumab procurement in Spain, or in other European tenders of biosimilars (Vogler et al., 2017). Although consensus on technical specifications remains pending, most of the literature agrees that tenders should avoid “price-only” criteria (as stated in the EU Directive itself (European Union, 2014b)) as well as “winner-takes-all” awards, or any combination of the two because awarding the entire market share to a single winner excludes other competitors for the duration of the contract, disincentivizes further investment in the area and potentially reduces the number of suppliers in the market. This can risk disrupting patient care continuity in the event of product shortages and ultimately leads to future monopolistic situations which could drive companies out of the market with potential future increase in prices that jeopardize savings made to date (Dranitsaris et al., 2017; Barbier et al., 2021; Barbier et al., 2022; Vogler et al., 2017).

**TABLE 2:** Characteristics of the evaluated tenders.

Autonomous community	Year	Tender reference	Estimated value of the contract (€)	Relevant estimated volume of the tender (with extension)	Maximum bidding price/DDD (€)	Price offered/DDD (€)	Price of the originator product/DDD (€)	Nº of successful bidders	Trade name of the product awarded (40 mg)
ANDALUSIA	2019	6TKBEAE	30,733,254	67,400	414.53	98.95	290.17	ONE	Imraldi^®^
ANDALUSIA	2020	6L3QLHB	7,524,000	36,000	190	60	188.10	ONE	Hyrimoz^®^
ARAGÓN	2020	62 DG/20	34,239,381	155,703	219.20	42	200	ONE/MULTIPLE The bidder with the highest score prevails However, for reasons of clinical, operational, or therapeutic convenience, the award may be granted to another successful bidder. This decision must be justified with a thoroughly documented report	Hyrimoz^®^
ASTURIAS	2019	SC_2019_012	23,851,260	147,230	135	98	290.17	ONE	Hulio^®^
BASQUE COUNTRY	2019	G/100/20/1/0091/OSC1/0000/012019	11,010,179	51,300	207.27	103	207	ONE	Imraldi^®^
BASQUE COUNTRY	2022	2022/02081	13,159,680	280,000	39	35		ONE	Imraldi^®^
CANARY ISLANDS	2019	23/S/19/SU/DG/A/AM008	37,703,529	129,936	290.17	79	280	ONE/MULTIPLE The bidder with the highest score prevails However, for reasons of clinical, operational, or therapeutic convenience, the award may be granted to another successful bidder. This decision must be justified with a thoroughly documented report	Hyrimoz^®^
CANARY ISLANDS	2024	23/S/23/SU/DG/A/AM15	39,342,996	301,680	125.40	43,50	125.40		Hyrimoz^®^
CATALONIA	2019	CSC F 13/18	38,631,402	80,136	459.63	129	331.62	FIVE The two bidders with the highest score prevail However, for reasons of clinical, operational, or therapeutic convenience, the award may be granted to the other successful bidders. This decision must be justified with a thoroughly documented report	Imraldi^®^
CATALONIA	2021	ICS/CC00/1101244000/21/MAR	12,898,640	130,472	70	42		MULTIPLE	Idacio^®^
CATALONIA	2022	CSC F 1/21	10,946,832	120,267	70	38		ONE/MULTIPLE The bidder with the highest score prevails However, for reasons of clinical, operational, or therapeutic convenience, the award may be granted to another successful bidder. This decision must be justified with a thoroughly documented report	Idacio^®^
CATALONIA	2024	CSC F 14/23	13,681,580	216,980	50	37			Idacio^®^
MADRID	2019	PA-SUM-45/2018	109,623,092	349,452	313,7	48	200		Idacio^®^
MURCIA	2023	CS/9999/1101097086/AM/2023	36,310,911	220,000	156,750	42	156,75	MULTIPLE All companies that meet the minimum requirements outlined in the bidding documents and whose price does not exceed the bid amount	Idacio^®^
Several (INGESA)	2022	2021/064	88,906,87	472,657	188,1	36,8		ONE/MULTIPLE The bidder with the highest score prevails However, for reasons of clinical, operational, or therapeutic convenience, the award may be granted to another successful bidder. This decision must be justified with a thoroughly documented report	Idacio^®^
VALENCIAN COMMUNITY	2021	199/2021	27,690,847	317,313	77	38			Idacio^®^

Estimated value of the contract (€) is calculated by multiplying the planned units to be purchased by the maximum bid price, taking into account any possible extensions stipulated in the contract plus any possible modifications to the contract. Relevant estimated volume is the estimated number of units to be consumed (including possible extensions). Maximum bidding price/DDD (€) is the price established in the tender in order to calculate the estimated value, which cannot be exceeded by the companies in order to participate in the tender. Price offered/DDD (€) is the price offered by the best bidder in the tender. Price of the originator product/DDD (€) is the price offered by the originator (if it submitted a bid).

Thus, our analysis reveals that although there is widespread adherence to the broad principles of EU directives (European Union, 2014b; European Union, 2014a), the methods of tendering and the variety of criteria followed in the public purchasing for adalimumab does not seem to adhere to a clear logic, even within a relatively homogeneous legal framework like that present in Spain (Ehlers et al., 2022). Given the intrinsic complexity of any tender, we are not suggesting that the system is characterized by bad governance or poor procurement practices. However, the broad disparity observed in the requirements set for the tender of a single drug within the same national territory raises the specter of unnecessary demands or bureaucratic overreach. This scenario complicates the actions of pharmaceutical companies and creates disparities in the availability of treatments for patients (Gawronski et al., 2022). Moreover, while considering the specific circumstances of Spain, this situation may be present in other countries and may be influenced by the novel and expanding nature of the European market for biosimilars (Barbier et al., 2021; Vogler et al., 2017; Kanavos et al., 2009). For instance, a recent study highlighted that purchasers, such as hospital pharmacists, often find it challenging to identify criteria beyond price when selecting between available off-patent biologics and new biosimilars with the correct formulation (Barbier et al., 2021). There is currently an insufficient volume of studies analyzing how other European markets handle the selection and evaluation of quality criteria in drug tenders, but research is beginning to emerge from both academia (Dranitsaris et al., 2017; Barbier et al., 2021; Barbier et al., 2022; Kanavos et al., 2009), and industry stakeholders (Gawronski et al., 2022; European Federation of Pharmaceutical Industries and Associations, 2022) proposing ways to address this gap by reaching consensus in which all stakeholders—industry, government, and clinicians—should be involved. While a standardized tender process may be unattainable, the development of more specific directives for contracts in particular sectors, including pharmaceutical products, seems necessary.

## 4 Conclusion

Although there is widespread adherence to EU directives (European Union, 2014b; European Union, 2014a), the methods of tendering for pharmaceutical products, represented here by a focus on the example of the monoclonal antibody adalimumab, and the variety of criteria followed do not seem to adhere to a clear logic within a relatively homogeneous legal framework like that of Spain.

## Data Availability

The original contributions presented in the study are included in the article/supplementary material, further inquiries can be directed to the corresponding author.
